# Associations of rheumatoid arthritis and rheumatoid factor with mental health, sleep and cognition characteristics in the UK Biobank

**DOI:** 10.1038/s41598-022-22021-6

**Published:** 2022-11-18

**Authors:** Ioana Stanciu, Jana Anderson, Stefan Siebert, Daniel Mackay, Donald M. Lyall

**Affiliations:** 1 School of Health and Wellbeing, University of Glasgow, 1 Lilybank Gardens, Glasgow, G12 8RZ UK; 2 School of Inflammation and Immunity, University of Glasgow, Glasgow, UK

**Keywords:** Rheumatoid arthritis, Sleep, Cognitive ageing, Epidemiology, Anxiety, Depression

## Abstract

While previous rheumatoid arthritis (RA) studies have focussed on cardiometabolic and lifestyle factors, less research has focussed on psychological variables including mood and cognitive health, and sleep. Cross-sectional analyses tested for associations between RA and RF+ (positive rheumatoid factor) vs. mental health (depression, anxiety, neuroticism), sleep variables and cognition scores in UK Biobank (total n = 484,064). Those RF+ were more likely to report longer sleep duration (*β* = 0.01, *SE* = 0.004, p < 0.01) and less likely to get up in the morning easily (OR 0.95, 95% CI 0.92–0.99, p = 0.01). Those reporting RA were more likely to score higher for neuroticism (*β* = 0.05, *SE* = 0.01, p < 0.001), to nap during the day (OR 1.10, 95% CI 1.06–1.14, p < 0.001), have insomnia (OR 1.28, 95% CI 1.22–1.35, p < 0.001), have slower reaction times (*β* = 0.02, *SE* = 0.008, p < 0.005) and score less for fluid intelligence (*β* = − 0.03, *SE* = 0.01, p < 0.05) and less likely to get up easily (OR 0.61, 95% CI 0.58–0.64, p < 0.001). The current study suggests that prevalent RA, and RF+ status are associated with differences in mental health, sleep, and cognition, highlighting the importance of addressing these aspects in clinical settings and future research.

## Introduction

Rheumatoid arthritis (RA), the most prevalent chronic inflammatory condition affecting the joints^1^, is characterized by pain, stiffness and swelling, which if not controlled, can lead to permanent damage. The estimated prevalence for RA in UK adults is around 0.81%, with a higher prevalence for women (1.16%) than for men (0.44%)^2^. Rheumatoid factor (RF) is an autoantibody that is present in approximately 80% of people with RA and can occur years before any clinical symptoms^3^. It can lead to worse outcomes, including higher disease activity and radiographic progression^4,5^. However, it can also be present in the general population^6^ including with multimorbidity^7^. If left untreated, RA can result in several negative outcomes including increased mortality, hospitalization, work disability and decreased quality of life^8^. RA can also be associated with other comorbidities, most notably cardiovascular disease^9^, mental health, sleep and cognitive problems.

### Mental health

Patients with RA are more likely to suffer from depression, even after controlling for age and sex^10^. Prevalence rates for depression in research studies vary considerably and can range between 0.04 and 66.3% depending on the instrument used for diagnosis^11^. The overall quality of published papers is also poor, with a median quality score of 3/10 and 82% of papers scoring 5/10 or lower^11^. An important issue with depression research in RA is that the two disorders share several symptoms such as fatigue and sleep disturbance that can often be excluded from analyses^12^ resulting in an underestimation of true prevalence rates. Nevertheless, even when the gold standard of diagnostic criteria for depression is used (i.e. DSM diagnostic criteria) prevalence rates for depression in RA are still high (16.8%)^11^ compared to prevalence estimates for the general population (4.1% for 1-year estimates and 6.7% for lifetime prevalence)^13^. Anxiety in RA has received considerably less attention but prevalence rates between 13.5 and 70% have been reported^14–16^. Also, a high neuroticism score at baseline was significantly associated (p < 0.01) with depression and anxiety at the 3 and 5-year follow up in RA patients, making it the most consistent and effective predictor of mental health in the study^17^. While Isik et al.^16^ reported no significant differences in anxiety and depression between RF+ and RF− groups, in another study people that were RF− spent twice as much time being treated for depression and were more likely to need longer depression treatment as a result of a prolonged diagnosis delay^18^. Ho et al.^19^ reported RF to be a significant predictor for severity of depression even after adjusting for confounding factors, while for anxiety, RF was a significant predictor only in the univariate analysis.

### Sleep

Poor sleep in RA is estimated to be present in at least 49% of patient cohorts, with many studies reporting figures as high as 80% for the RA subsample^20–23^. Zhang et al.^24^ undertook the first meta-analysis (n = 1143) evaluating poor sleep in RA as measured by the PSQI (Pittsburgh Sleep Quality Index) and suggested that RA patients scored higher (indicative of worse sleep) than the healthy control group in every domain of the questionnaire (i.e. subjective sleep quality, sleep latency, sleep duration, habitual sleep efficiency, presence of sleep disorders, use of sleeping medication and daytime dysfunction as a consequence of poor sleep) and total PSQI score. When comparing between poor sleepers with RA and good sleepers with RA, poor sleepers are more likely to have higher disease activity scores, worse night pain and total pain and higher rates for depression and anxiety, while the good sleep RA subsample reported using a significantly higher percentage of synthetic DMARDs (Disease-modifying antirheumatic drugs)^22^. In contrast, RF status did not significantly differ between poor and good sleepers^21,22^.

### Cognition

A recent systematic review including 15 papers and 749 participants has suggested that the RA patient-population might show cognitive differences that could warrant attention in clinical and research settings, but more studies are needed to better understand prevalence rates and what specific cognitive domains are impaired^25^. RA patients either had worse performances than healthy controls on average or performed at a level that was below their age-related norms. Considering that some of the studies included also had other comparison clinical groups (i.e. fibromyalgia, systemic lupus erythematosus, Sjögren’s syndrome) that also had worse performances at cognitive tasks, the authors suggest that cognitive impairment may be present in other chronic, inflammatory or autoimmune conditions involving pain. Cognitive impairment on a range of cognitive tests ranged from 8% in the semantic fluency test (recall test) to 29% in the design fluency test, with 30% of RA patients displaying impairment in four or more tests (n = 122, mean age = 58.4 ± 10.8)^26^. In the multivariable analyses, use of glucocorticoids and cumulative number of cardiovascular disease (CVD) factors were independently associated with cognitive impairment in this patient population. Regarding individual components of cognition, Vitturi et al.^27^ observed significantly worse performance (*p* < 0.001) in RA for the attention, remote memory, repetition, stage command, writing, read and obey and copy tasks. Those using biologics were less likely to be classified as cognitively impaired using the MMSE (Mini-mental state examination) (p = 0.05), while those under glucocorticoids and that were RF+ were more likely to be classified as cognitively impaired, according to the MoCA (Montreal Cognitive Assessment) (p = 0.01 and = 0.05 respectively).

Despite being important contributors to quality of life, sleep, mental health, and cognition have been largely neglected in the RA literature. Most studies to date had a sample size of under 1000 people and use different instruments to test these associations, without properly controlling for covariates. As the systematic review carried out by Matcham et al.^11^ pointed out there were even 40 different ways to define depression as well as to measure it leading to different prevalence rates and an overall poor quality of published papers. Similarly, there is a lack of understanding and a huge gap in the literature about the role of rheumatoid factor positivity (RF+) on these domains. We aim to provide a solution to these problems by using a very large population cohort of around 500,000 participants all measured identically, by looking at both self-reported rheumatoid arthritis and rheumatoid factor and by controlling for a wide range of covariates. In conclusion, the aim of the current study was to characterize mental health, cognition, and sleep variables in people with RA and to compare these associations in people with positive RF (RF+) and negative RF (RF−) in a large population cohort.

## Methods

### Participants and procedure

The current study used the UK Biobank, a large database that contains an extensive range of health measures for around half a million participants in the UK. People aged between 40 and 69 years old that were registered with the National Health Service (NHS) and lived up to approximately 25 miles from one of the 22 UK Biobank assessment centers were invited to take part^28^. Around 5.47% agreed-equivalent to around 500,000 participants- and recruitment took place between 2006 and 2010. All volunteers gave informed consent, and the study was conducted under generic approval from the NHS National Research Ethics Service (approval letter dated 17th June 2021, Ref 11/NW/0382) and under UK Biobank project approval 17689 (PI Lyall). All methods were performed in accordance with relevant guidelines and regulations, informed consent was collected from every participant and the data was anonymized. At the baseline visit at the assessment centre participants completed touchscreen questionnaires on their sociodemographic factors (such as age, sex, ethnicity, postcode of residence), lifestyle (e.g. smoking status, alcohol intake), medical history (including mental health and musculoskeletal problems) and had several physical measures taken and samples of blood, saliva and urine collected^28^. Area-based socioeconomic deprivation was measured using Townsend scores derived from postcode of residence^29^. In the current study RA, depression, anxiety and cardiometabolic diseases are based on self-report.

All UK Biobank participants had biomarker levels measured at baseline from serum and packed red blood cell samples. For the current study, very low levels of rheumatoid factor that were coded as ‘missing’ in the original data were recoded conservatively as the square root of the minimum stated detectable value if participants had data for a remaining biomarker^30^. A new binary variable was then computed for rheumatoid factor category with variables over 14 IU/ml considered rheumatoid factor positive (RF+)^31^. We removed those that failed biomarker quality control (coded as ‘no data returned’ or logged as having unrecoverable aliquot problems). Details about biomarker quality control, measurements and analysis methods can be found at:

https://www.ukbiobank.ac.uk/media/oiudpjqa/bcm023_ukb_biomarker_panel_website_v1-0-aug-2015-edit-2018.pdf,

https://biobank.ndph.ox.ac.uk/showcase/showcase/docs/haematology.pdf,

https://biobank.ndph.ox.ac.uk/showcase/showcase/docs/serum_biochemistry.pdf, and

https://biobank.ndph.ox.ac.uk/showcase/showcase/docs/biomarker_issues.pdf.

At the assessment centre, five cognitive tests were administered to participants via a touchscreen computer. It took approximately 15 min to complete all tests (numeric memory excluded in the present study due to low completion rates^32^. For reaction time (N = 496,771), mean response time (milliseconds) was recorded to matching pairs of visual stimuli. The fluid intelligence/verbal-numeric reasoning touchscreen assessment (N = 209,473) was comprised of questions designed to assess the capacity to solve problems that require logic and reasoning ability, independent of acquired knowledge. Prospective memory (N = 216,124) tested the ability for the participant to remember and follow instructions given before a task. The data used for the present analyses was dichotomized as correctly recalled on first attempt or not. The pairs matching task (N = 498,748) assessed visuo-spatial memory. For this paper, the used variable was the number of errors made.

At the assessment centre participants also completed touchscreen questionnaires about their lifestyle and environment, including mental health (depression, anxiety, derived summary score of neuroticism based on 12 neurotic behaviour domains) and sleep variables such as sleep duration (h/day), frequency of napping during the day (ordinal with 4 options), ease of getting up in the morning (ordinal with 6 options) and insomnia frequency (ordinal with 4 options). Multi-level ordinal variables were dichotomized for the current study: ever nap during the day (No/Yes), ease of getting up in the morning (Not easy/Easy) and insomnia (No/Yes).

### Statistical analyses

We used baseline data from the UK Biobank cohort (n = 502,506) to cross-sectionally compare people with and without RA, and people that are RF+ versus RF− on a variety of sociodemographic, lifestyle, illness-related factors and mental health, performance on cognitive tests and sleep-related factors^33^. We performed logistic and linear regression analyses to determine whether RF+ status (i.e. seropositivity) or RA diagnosis were associated with mental health, cognition, and sleep variables. More specifically, we performed linear regression models for the neuroticism, sleep duration, reaction time, fluid intelligence and pairs matching variables and logistic regressions for the depression, anxiety, nap during the day, getting up in the morning, insomnia, and prospective memory variables. We adjusted for the covariates of age, sex, self-reported ethnicity, deprivation index, smoking status, BMI and alcohol intake (frequency). We removed those that were advised by their doctor to stop drinking alcohol and recorded those with a BMI under 18 as missing. For sleep analyses we also controlled for cardiometabolic diseases.

## Results

### Demographics

Of the approximately 500,000 baseline UK Biobank participants, 5722 (1.18%) self-reported having RA and 25, 772 (5%) were RF+ (see Table 1). Out of those that self-reported RA 74% were RF− and 26% were RF+. For the current study we have split the baseline sample between self-report RA and non-RA and RF+ versus RF− to compare any differences between these subsamples. Participants that reported RA were older and more likely to be female (69% of RA patients compared to 31% for males). The RA subsample was more likely to report being a current or previous smoker but more likely to drink alcohol less frequently. They were also more likely to have higher values for BMI, hip circumference, and waist circumference. Participants with RF+ were also older and more likely to be female (56% compared to 44% males). There were also significant differences between RF− and RF+ for ethnicity, smoking status, alcohol intake, deprivation index and physical activity score.

**Table 1:** Characteristics of participants with/without rheumatoid arthritis and by rheumatoid factor seropositivity.

	No rheumatoid arthritis n = 484,064	Self-reported rheumatoid arthritis n = 5722	p value	Negative rheumatoid factor (RF−) n = 463,415	Positive rheumatoid factor (RF+) n = 25,772	p value
Age^a,c^	58 (50–63)	61 (55–65)	< 0.001	58 (50–63)	60 (53–64)	< 0.001
**Sex**^**b,d**^						
Female	261,960 (54%)	3829 (69%)	< 0.001	251,476 (54%)	14,413 (56%)	< 0.001
Male	222,104 (46%)	1793 (31%)		212,495 (46%)	11,402 (44%)	
**Ethnicity**^**b,d**^						
White British	456,123 (94.3%)	5394 (94.4%)	0.8	437,102 (94.3%)	24,415 (94.7%)	0.05
Other	21,321 (4.4%)	251 (4.4%)		10,497 (2.2%)	574 (2.2%)	
Missing	6110 (1.2%)	67 (1.1%)		5875 (1.3%)	302 (1.2%)	
**Smoking**^**b,d**^						
Never	264,224 (55%)	2691 (47%)	< 0.001	253,343 (55%)	13,572 (53%)	< 0.001
Previous	166,767 (35%)	2273 (40%)		159,593 (35%)	9447 (37%)	
Current	50,625 (11%)	710 (13%)		48,675 (10.5%)	2660 (10%)	
**Alcohol**^**b,d**^						
Daily/almost daily	98,894 (20.4%)	840 (14.5%)	< 0.001	93,984 (20.3%)	5750 (22.3%)	< 0.001
3–4 times/week	112,175 (23.2%)	967 (16.8%)		107,269 (23.1%)	5873 (22.7%)	
1–2 times/week	124,962 (25.8%)	1429 (25%)		120,020 (25.9%)	6371 (24.7%)	
1–3 times/month	53,834 (11.1%)	689 (12%)		51,795 (11.2%)	2728 (10.6%)	
Special occasions	55,442 (11.5%)	945 (16.5%)		53,447 (11.5%)	2940 (11.4%)	
Never	37,682 (7.8%)	839 (14.6%)		36,418 (7.8%)	2103 (8.1%)	
BMI^a,c^	26.74 (24.2–29.9)	27.5 (24.5–31.1)	< 0.001	26.7 (24.2–29.9)	26.7 (24.1–29.9)	0.44
Hip circumference^a,c^	102 (97–108)	103 (98–110)	< 0.001	102 (97–108)	102 (97–108)	0.6
Waist circumference^a,c^	90 (80–99)	91 (81–101)	< 0.001	90 (80–99)	90 (80–99)	0.1
**Deprivation quintile**^**b,d**^						
1 (least deprived)	167,073 (34.5%)	1736 (30.1%)	< 0.001	159,717 (34.4%)	9092 (35.2%)	0.001
2	103,801 (21.4%)	1160 (20.3%)		99,359 (21.4%)	5602 (21.7%)	
3	80,312 (16.6%)	906 (15.9%)		76,975 (16.6%)	4243 (16.5%)	
4	73,770 (15.3%)	1001 (17.5%)		70,891 (15.3%)	3880 (15.1%)	
5 (most deprived)	58,519 (12.1%)	909 (15.9%)		56,473 (12.2%)	2955 (11.5%)	
Physical activity score^a,c^	520 (270–1020)	550 (270–1080)	0.1	520 (270–1020)	510 (267–990)	< 0.05

^a^Kruskal-Wallis test.

^b^Pearson χ^2^ test.

^c^Median (IQR).

^d^N (%).

### Associations

#### Mental health

In the unadjusted regression models, people RF+ were more likely to score less for neuroticism (*β* = − 0.01, *SE* = 0.005, p < 0.001) (see Supplementary Table S2), while people that reported RA were more likely to have depression (OR 1.16, 95% CI 1.08–1.25, p < 0.001) and score higher for neuroticism (*β* = 0.08, *SE* = 0.01, p < 0.001) (see Table 2). After adjusting for covariates, those that reported RA were still more likely to score high for neuroticism (*β* = 0.05, *SE* = 0.01, p < 0.001) but less likely to report anxiety (OR 0.80, 95% CI 0.67–0.95, p < 0.01).

**Table 2:** Regression models for the association between RA and mental health.

	Unadjusted model			Fully adjusted model^c^		
	β/OR	95% CI	P value	β/OR	95% CI	P value
Depression^a^	1.16	1.08–1.25	< 0.001	1.03	0.95–1.10	0.4
Anxiety^a^	0.88	0.74–1.04	0.16	0.80	0.67–0.95	0.01
Neuroticism^b^	0.08 (0.01)	0.06–0.10	< 0.001	0.05 (0.01)	0.03–0.07	< 0.001

^a^Logistic regression with OR.

^b^Linear regression with standardized betas and SE.

^c^Adjusted for the covariates of age, sex, ethnicity (White British vs Other), deprivation index, smoking status, BMI and alcohol intake.

#### Sleep

Regarding sleep, those RF+ were more likely to report longer sleep durations (*β* = 0.01, *SE* = 0.004, p < 0.001), more likely to nap during the day (OR 1.06, 95% CI 1.03–1.09, p < 0.001), and more likely to have insomnia (OR 1.06, 95% CI 1.03–1.10, p < 0.001) (see Supplementary Table S3) while those that self-reported RA were more likely to nap during the day (OR 1.21, 95% CI 1.17–1.26, p < 0.001), less likely to get up in the morning with ease (OR 0.61, 95% CI 0.59–0.63, p < 0.001) and more likely to have insomnia (OR 1.48, 95% CI 1.41–1.56, p < 0.001) (see Table 3). After adjusting for covariates, those RF+ were more likely to report longer sleep duration (*β* = 0.01, *SE* = 0.004, p < 0.01) and less likely to get up in the morning easily (OR 0.95, 95% CI 0.92–0.99, p = 0.01). Those self-reporting having RA were more likely to nap during the day (OR 1.10, 95% CI 1.06–1.14, p < 0.001), less likely to get up with ease (OR 0.61, 95% CI 0.58–0.64, p < 0.001) and more likely to suffer from insomnia (OR 1.28, 95% CI 1.22–1.35, p < 0.001).

**Table 3:** Regression models for the association between RA and sleep.

	Unadjusted model			Fully adjusted model ^c^		
	β/OR	95% CI	P value	β/OR	95% CI	P value
Sleep duration^b^	0.01 (0.009)	− 0.02 to 0.007	0.2	− 0.01 (0.009)	− 0.03 to 0.001	0.06
Nap during the day^a^	1.21	1.17 to 1.26	< 0.001	1.10	1.06 to 1.14	< 0.001
Getting up in the morning^a^	0.61	0.59 to 0.63	< 0.001	0.61	0.58 to 0.64	< 0.001
Insomnia^a^	1.48	1.41 to 1.56	< 0.001	1.28	1.22 to 1.35	< 0.001

^a^Logistic regression with OR.

^b^Linear regression with standardized betas and *SE.*

^c^Adjusted for the covariates of age, sex, ethnicity (White British vs Other), deprivation index, smoking status, BMI, alcohol intake and cardiometabolic diseases.

#### Cognition

For cognition, those RF+ were more likely to score higher for reaction time (*β* = 0.03, *SE* = 0.004, p < 0.001) (see Supplementary Table S4). Those that self-reported having RA were also more likely to score higher for reaction time (*β* = 0.13, *SE* = 0.009, p < 0.001), but more likely to score less for fluid intelligence (*β* = − 0.10, *SE* = 0.01, p < 0.001) and less likely to perform well on the prospective memory task (OR 0.87, 95% CI 0.81–0.94, p < 0.001) (see Table 4). After adjusting for covariates, reaction time (*β* = 0.02, *SE* = 0.008, p < 0.005) and fluid intelligence (*β* = − 0.03, *SE* = 0.01, p < 0.05) remained significant predictors for RA.

**Table 4:** Regression models for the association between RA and cognition.

	Unadjusted model			Fully adjusted model ^c^		
	β/OR	95% CI	P value	β/OR	95% CI	P value
Reaction time^b^	0.13 (0.009)	0.11 to 0.14	< 0.001	0.02 (0.008)	0.009 to 0.04	0.002
Fluid intelligence^b^	− 0.10 (0.01)	− 0.13 to −0.07	< 0.001	− 0.03 (0.01)	− 0.07 to − 0.004	0.02
Pairs matching^b^	0.008 (0.02)	− 0.03 to 0.05	0.7	− 0.02 (0.021)	− 0.06 to 0.01	0.2
Prospective memory^a^	0.87	0.81 to 0.94	< 0.001	0.97	0.90 to 1.05	0.5

^a^Logistic regression with OR.

^b^Linear regression with standardized betas and *SE.*

^c^Adjusted for the covariates of age, sex, ethnicity (White British vs Other), deprivation index, smoking status, BMI and alcohol intake.

## Discussion

The prevalence of self-report RA within the UK Biobank baseline sample was 1.18%, out of which 69% were female and 31% were males. This is similar to other estimates in the literature that suggest around 0.81% of people in the UK suffer from RA, with a higher prevalence for women than men^2^. Out of the 5722 participants that reported RA, 74% were RF− and 26% were RF+. This was much lower than previous estimates of 80%^3^ and it could be due to differences in measurement, measurement error or changes in RF levels compared to previous population recorded values. RA patients were more likely to report being a current or previous smoker, in line with previous literature suggesting smoking is a risk factor for developing RA^34^. There were also significant differences between RF+ and RF− people regarding smoking as Sugiyama et al.^34^ suggested that odds of having RA are almost double for RF+ male smokers. The RA subsample was more likely to drink alcohol less frequently. Observational studies often suggest a ‘protective’ effect of alcohol for RA but this can be due to confounding recall bias^35^. It is possible that those with more severe RA drink less alcohol or that those that completely abstain from drinking do so due to medical reasons and are less healthy overall compared to those that drink occasionally. Indeed, Mendelian randomization analyses generally do not support the inverse causal relationship between alcohol and RA^36^. The RA subsample was also more likely to have higher values for BMI, hip circumference, and waist circumference. Having a BMI over 30 was associated in the literature with an increased risk of developing RA (RR = 1.25, 95% CI 1.07–145)^37^ and 40% lower odds of achieving remission^38^. To our knowledge there is a gap in the literature comparing people RF− and RF+ but we found significant differences for age, sex, ethnicity, smoking status, alcohol intake, deprivation, and physical activity score (but not BMI).

Among RA comorbidities, cardiovascular-related disease was studied most extensively as it is believed that RA patients have a 50% increased risk of CVD-related mortality compared to the general population (meta-SMR 1.50, 95% CI 1.39–1.61). Even after accounting for traditional cardiovascular risk factors, there is still a large proportion of heart failure (at the age of 80) left unexplained in RA patients (*p* < 0.01)^39^. The RA subsample in the current study was also more likely to report diabetes, hypertension, heart attack and stroke, while RF+ people were more likely to report hypertension, heart attack and stroke (see Supplementary Table S1).

Regarding mental health, there were significant differences between RA and non-RA groups on average, and RF+ vs RF− for depression and neuroticism. The prevalence rate for depression in RA was 6.9% and for anxiety was 1.1% (see Supplementary Table S1). Prevalence rates for depression in the literature can range between 0.04 and 66.3% depending on the instrument used for diagnosis^11^. The current study was limited to using self-report depression and anxiety. After adjusting for covariates, RA people were more likely to score higher for neuroticism and less likely to report anxiety. RA was associated with higher neuroticism in our analyses, even after adjusting for covariates. This can be essential for mental health in RA as Evers et al.^17^ suggested neuroticism is the most consistent and effective predictor of mental health issues in RA at the 3 and 5 years follow up. Regarding sleep, previous studies estimated that between 49 and 80% of RA patients report poor sleep^20–23^. In our study, those RF+ were more likely to report longer sleep duration and less likely to get up in the morning with ease, even after adjusting for covariates. Those that reported having RA were more likely to nap during the day, less likely to get up with ease in the morning and more likely to suffer from insomnia even after adjusting for covariates. For cognition, a systematic review suggested that people with RA might have cognitive impairment but more research is needed to better understand what specific domains are affected^25^. In our study, RF+ people were more likely to score higher for reaction time only in the unadjusted regression analyses. In the unadjusted regressions those that reported having RA were more likely to score higher for reaction time and less for the fluid intelligence and the prospective memory tasks. After adjusting for covariates, reaction time and fluid intelligence remained significant predictors. Considering that RA is a disease that can cause joint stiffness and pain, we believe a reaction time task can be severely impacted by this, unveiling a problem with mobility rather than cognition.

The UK Biobank has the advantage of being a relatively large cohort with around half a million people recruited, tested, and measured in the same way. However, it might not be representative of the population for lifestyle risk factors and disease, and even age or deprivation; there is a well-established ‘healthy bias’ and some evidence this may meaningfully and significantly bias analyses towards type 1/2 errors^40,41^. It is possible that those with less severe RA or mental health take part in research studies. The current study was also limited to using self-report illness (e.g., depression, anxiety, and rheumatoid arthritis). As in a previous study by Siebert et al.^42^ this meant that we could not differentiate between participants that preferred not to report or did not remember their diseases, and those that were actually healthy and hence had nothing to report. However, our self-report RA prevalence was similar to previous estimates. Generally, choosing not to answer queries was relatively rare in UK Biobank at < 5%. We believe that the ‘getting up in the morning’ question should have been phrased differently as it does not differentiate between difficulties waking up and difficulties getting out of bed. People with RA suffer from joint stiffness, especially in the morning which could make it more difficult for them to get out of bed. Similarly, we observed an association with RF+ and poorer sleep; the exact mediating mechanisms underlying this are unclear. Future research, may investigate the possibility that joint deformities, higher chronic pain and other comorbidities may contribute to the observed associations. In our study, those RF+ were more likely to report longer sleep duration and more likely to have insomnia which may appear contradictory. The UK Biobank insomnia question was based on self-report and asked participants to include naps as well. We believe that naps should have been excluded from this question as they can give the impression of a long sleep duration at night when in fact quite the opposite could be true—a person could suffer from insomnia which would make them more likely to need a nap throughout the day. Future research should also investigate this further based on more objective sleep measures.

The cross-sectional nature of the current study means that we could not establish causal relationships and rule out confounding and reverse causation. It is unclear whether mental health, sleep and cognition predate or are caused by RA. By only using a single measurement of RF we were also unable to investigate whether levels fluctuate over time or whether they are stable. Future research could benefit from using primary care and hospital admissions diagnoses and genetic or longitudinal analyses for causal relationships.

Despite being important areas of RA patients’ overall quality of life, mental health, sleep and cognition are often overlooked in the literature and in the clinic. Similarly, we need a better understanding about the role RF plays on these domains. The current study suggests that self-reported RA and RF status are associated with differences in all three domains and provide substantial support to previous literature, highlighting the importance of addressing these aspects in clinical settings and future research.

## Supplementary Information

Supplementary Information.

## Data Availability

Data is available from the UK Biobank website for a fee via their data access procedure: https://www.ukbiobank.ac.uk/enable-your-research/register.
