# Supplementary material for: Associations of rheumatoid arthritis and rheumatoid factor with mental health, sleep and cognition characteristics in the UK Biobank
Source: Sci Rep. 2022 Nov 18;12:19844. doi: 10.1038/s41598-022-22021-6 (PMC9674828; doi:10.1038/s41598-022-22021-6)
Supplement: Supplementary file 1 — Supplementary Information. [file 41598_2022_22021_MOESM1_ESM.docx]

**Supplementary Table S1** Frequency of comorbid conditions between participants with/without rheumatoid arthritis and by rheumatoid factor seropositivity

|  | **No rheumatoid arthritis**  **n= 484 064** | **Self-reported rheumatoid arthritis**  **n= 5722** | **p-value** | **Negative rheumatoid factor (RF-)**  **n= 463 415** | **Positive rheumatoid factor (RF+)**  **n= 25 772** | **p-value** |
| --- | --- | --- | --- | --- | --- | --- |
| Depression (self-report)  ^b,d^ | 27 379 (5.7%) | 396 (6.9%) | <0.001 | 26 379 (5.7%) | 1396 (5.4%) | 0.06 |
| Anxiety (self-report) ^b,d^ | 6725 (1.4%) | 67 (1.1%) | 0.16 | 6459 (1.4%) | 333 (1.3%) | 0.17 |
| Neuroticism score ^a,c^ | 4 (1-6) | 4 (2-7) | <0.001 | 4 (1-6) | 4 (1-6) | 0.002 |
| Diabetes ^b,d^ | 20 866 (4.3%) | 364 (6.4%) | <0.001 | 20 128 (4.3%) | 1102 (4.2%) | 0.5 |
| Hypertension ^b,d^ | 125 782 (26%) | 1973 (34.5%) | <0.001 | 120 295 (26%) | 7460 (29%) | <0.001 |
| Heart attack ^b,d^ | 11 171 (2.3%) | 225 (4%) | <0.001 | 10 738 (2.3%) | 658 (2.5%) | 0.01 |
| Stroke ^b,d^ | 6508 (1.3%) | 143 (2.5%) | <0.001 | 6266 (1.3%) | 385 (1.5%) | 0.05 |

^a^ Kruskal-Wallis test

^b^ Pearson χ^2^ test

^c^ Median (IQR)

^d^ N (%)

**Supplementary Table S2** Regression models for the association between RF-/RF+ and mental health

|  | Unadjusted Model | | | Fully adjusted model ^c^ | | |
| --- | --- | --- | --- | --- | --- | --- |
|  | β/  OR | 95% CI | P value | β/  OR | 95% CI | P value |
| Depression ^a^ | 0.96 | 0.92-1.00 | 0.06 | 0.97 | 0.93-1.01 | 0.2 |
| Anxiety ^a^ | 0.94 | 0.87-1.02 | 0.172 | 0.95 | 0.88-1.03 | 0.2 |
| Neuroticism ^b^ | -0.01  (0.005) | -0.09- -0.02 | <0.001 | -0.006  (0.004) | -0.01-0.002 | 0.16 |

^a^ Logistic regression with OR

^b^ Linear regression with standardized betas and *SE*

^c^ *Adjusted for the covariates of age, sex, ethnicity (White British vs Other), deprivation index, smoking status, BMI and alcohol intake*

**Supplementary Table S3** Regression models for the association between RF-/RF+ and sleep

|  |  | Unadjusted model |  |  | Fully adjusted model ^c^ |  |
| --- | --- | --- | --- | --- | --- | --- |
|  | β/  OR | 95% CI | P value | β/  OR | 95% CI | P value |
| Sleep duration ^b^ | 0.01  (0.004) | 0.01-0.02 | <0.001 | 0.01  (0.004) | 0.003-0.020 | 0.008 |
| Nap during the day ^a^ | 1.06 | 1.03-1.09 | <0.001 | 1.01 | 0.98-1.04 | 0.2 |
| Getting up in the morning ^a^ | 1.02 | 0.99-1.06 | 0.09 | 0.95 | 0.92-0.99 | 0.01 |
| Insomnia ^a^ | 1.06 | 1.03-1.10 | <0.001 | 1.01 | 0.98-1.04 | 0.4 |

^a^ Logistic regression with OR

^b^ Linear regression with standardized betas and *SE*

^c^ *Adjusted for the covariates of age, sex, ethnicity (White British vs Other), deprivation index, smoking status, BMI, alcohol intake and cardiometabolic diseases*

**Supplementary Table S4** Regression models for the association between RF-/RF+ and cognition

|  |  | Unadjusted model |  |  | Fully adjusted model ^c^ |  |
| --- | --- | --- | --- | --- | --- | --- |
|  | β/  OR | 95% CI | P value | β/  OR | 95% CI | P value |
| Reaction time ^b^ | 0.03  (0.004) | 0.03-0.04 | <0.001 | 0.001  (0.004) | -0.006- 0.009 | 0.7 |
| Fluid intelligence ^b^ | 0.005  (0.007) | -0.01- 0.02 | 0.5 | 0.01  (0.007) | -0.003- 0.02 | 0.1 |
| Pairs matching ^b^ | 0.002  (0.009) | -0.016- 0.02 | 0.8 | -0.01  (0.009) | -0.03-0.003 | 0.1 |
| Prospective memory ^a^ | 0.98 | 0.95-1.02 | 0.4 | 1.01 | 0.97-1.05 | 0.4 |

^a^ Logistic regression with OR

^b^ Linear regression with standardized betas and *SE*

^c^ *Adjusted for the covariates of age, sex, ethnicity (White British vs Other), deprivation index, smoking status, BMI and alcohol intake*
